# Deregulation of MicroRNA-146a and 155 expression levels might underlie complicated pregnancy in *Toxoplasma Gondii* seronegative women

**DOI:** 10.1186/s12905-024-03233-6

**Published:** 2024-07-23

**Authors:** Marwa M. Naguib, Youssef Abdel Zaher, Hemat Salah M. Ali, Hamasat Abdelhafiz Elnoury, Lina Abdelhady Mohammed, Omnia Youssef Habashy, Dina Abdelhady Mohammed

**Affiliations:** 1 Department of Parasitology, Faculty of Medicine, Benha University, Benha, Egypt; 2 Department of Obstetrics & Gynecology, Faculty of Medicine, Benha University, Benha, Egypt; 3 Department of Clinical & Chemical Pathology, Faculty of Medicine, Benha University, Benha, Egypt; 4 Department of Biochemistry, Faculty of Medicine, Benha University, Benha, Egypt

**Keywords:** High-risk women, *T. Gondii* infection, Pregnancy outcomes, Serodiagnosis, Plasma microRNA levels

## Abstract

**Background:**

To evaluate the ability of the estimated plasma expression levels of genes of microRNA (MiR-) 146a and 155 to differentiate between samples of pregnant women suspected to be infected by *T. gondii*. 50 newly pregnant women who had at least one of the criteria of high risk for *toxoplasma* infection and 50 newly primigravida women free of these criteria gave blood samples for qualitative determination of serum *toxoplasma* antibodies and estimation of plasma expression levels of MiR-146a and 155 using the qRT-PCR. During the pregnancy course, the incidence of pregnancy complications was recorded.

**Results:**

Twenty-six women were IgM^−^/IgG^−^, 17 women were IgM^+^/IgG^−^ and 7 women were IgM^+^/IgG^+^. Thirty-two women had pregnancy complications with significantly lower incidence in IgM^−^/IgG^−^ women. Plasma expression levels of MiR-146a and 155 were significantly higher in total patients compared to control levels and were significantly higher in samples of IgM^+^/IgG^+^ patients than in other samples. Statistical analyses defined a high plasma level of MiR-155 as the highly significant predictor for oncoming pregnancy complications and high levels of both microRNAs as predictors for the presence of toxoplasmosis despite seronegativity. Kaplan-Meier regression analysis defined increasing cumulative risk of having toxoplasmosis despite seronegativity with plasma levels of MiR-146a and MiR-155 of 1.2 and 3, respectively.

**Conclusion:**

The incidence of pregnancy complications is high, irrespective of the seronegativity of women at high risk of toxoplasmosis. Estimated plasma levels of MiR-155 might identify women liable to develop complications and differentiate seronegative women vulnerable to having *T. gondii* infection.

**Trial registration:**

The study protocol was approved preliminarily by the Local Ethical Committee at Benha Faculty of Medicine. Before enrollment, the study protocol was discussed in detail with the study participants, and those accepted to participate in the study signed written fully informed consents. The final approval of the study protocol was obtained after the end of case collection and registered by RC: 5-11-2022.

## Background

*Toxoplasma gondii* (*T. gondii*) is an obligate protozoan parasite that might be responsible for the development of several neurodegenerative manifestations and certain death for human hosts in special cases [1]. *T. gondii* belongs to the phylum *Apicomplexa*, which is characterized by high rate of growth and replication, thus requires nutrients that were obtained by scavenging from the host cell or by the de novo synthesis [2]. *T. gondii* has complex metabolic machinery for the acquisition of metabolites [3] and depends on cyclic GMP-dependent signaling to trigger its timely egress from host cells in response to various signals [4].

The process of placentation to create the syncytiotrophoblasts, which is a cell type at the maternal-fetal interface, is essential for normal pregnancy progress and favorable outcomes [5]. Various pregnancy adverse outcomes are mainly due to defective placentation process, apoptosis of syncytiotrophoblasts [5], disturbed immune milieu or metabolic environment at the maternal-fetal interface [6].

Infection by *T. gondii* early in pregnancy may induce spontaneous abortion and fetal malformation [7] and can induce maternal, fetal, and neonatal complications [8]. Maternal exposure to nonchemical stressors as inflammatory, immune, or endocrine, is associated with epigenetic and/or genomic changes in a tissue-specific manner and mediates certain pathways to modulate perinatal health outcomes [9]. Recent studies linked the *T. gondii*-induced pregnancy adverse outcomes to dysregulation of the expression levels of genes encoding for microRNAs [10]. The microRNAs (MiRs) are small non-coding short-nucleotide chains, both the circulating [11] and placental [12] microRNAs may function in the development of adverse pregnancy and newborn health outcomes.

Positive serum immunoglobulin G (IgG) against toxoplasmosis was used to determine the presence of infection and estimation of serum immunoglobulin M (IgM) could help to estimate the time of infection [13]. However, a recent study detected *T. gondii* DNA in seronegative subjects and recommended supplementing routine serological testing with a molecular method [14].

## Methods

### Objectives

The present study tried to evaluate the ability of the estimated plasma expression levels of genes of MiR-146a and 155 to discriminate samples of infected women among *T. gondii* seronegative pregnant women and to predict pregnancy outcomes.

### Design

A prospective observational case-control study.

### Blindness

Blood samples were collected by an assistant at the Antenatal Care Unit (ANC), University Hospital in clean dry tubes and labeled by the number of the patient according to enrolment schedules and sent for investigations as innominate, numbered tubes. The parasitologist was blinded to the clinical data and the results of molecular analyses. The biochemist was also blinded about the clinical indication for the requested analyses and the results of the parasitological examinations. The obstetrician was blinded about the results of the requested investigations till the end of case collection and determination of pregnancy outcomes. At the end of the study, the collected clinical data, pregnancy outcomes, and laboratory investigations were gathered and interpreted.

### Study participants

All women attending the ANC unit for assurance of being pregnant after having a missed period were evaluated for chemical pregnancy and those who had positive pregnancy tests gave blood samples for laboratory investigations, underwent clinical evaluation, and were asked to re-attend the ANC unit for assurance of pregnancy by detection of the presence of gestational sac using abdominal ultrasonography.

Clinical evaluation entails the collection of demographic data including age, weight, and height for calculation of baseline body mass index (BMI = weight in kg/height in m^2^), level of education, residence place, type of work, the possibility of contact with pets, or large animals or their excreta and socioeconomic status. History taking included the presence of previous complicated pregnancy, positive serological tests for toxoplasmosis, the presence of medical disorders, and the presence of special eating habits. Clinical examination to determine baseline systolic and diastolic blood pressures (SBP & DBP), fasting and postprandial blood glucose (FBG & PPBG), and evaluation for the presence of insulin resistance (IR) that was diagnosed if HOMA-IR score was > 2.

### Exclusion criteria

Previous history of positive serology for toxoplasmosis, complicated pregnancy, manifest diabetes mellitus or essential hypertension, medical diseases especially renal diseases associated with proteinuria.

### Inclusion criteria

All newly pregnant women who had at least one of the criteria of high risk for toxoplasmosis infection that were previously documented by CDC [13], accepted to participate in the study and were free of exclusion criteria were included. The study included an equal number of newly primigravida women free of inclusion and exclusion criteria to give blood samples at the time of diagnosis of pregnancy as a control group for levels of microRNAs.

### Blood sampling

Blood samples were collected at the time of chemical diagnosis of pregnancy and were divided between two dry clean tubes: the 1st tube was an EDTA-containing tube for obtaining plasma and was kept frozen at -80^o^C till estimation of the plasma expression levels of MiR-146 and 155 using the quantitative reverse transcriptase polymerase chain reaction (qRT-PCR). The 2nd part of the sample was allowed to clot and centrifuged for 15-min at 3000 rpm to separate serum and was kept frozen at -80^o^C till being ELISA assayed for detection of IgM and IgG.

### Investigations

1. ELISA qualitative determination of serum *Toxoplasma* IgM and IgG antibodies by enzyme-linked immunosorbent assay kits (ELISA kits, Abcam Inc., San Francisco, USA; Cat No. 108,778 and 108,776) according to the manufacturer’s instructions and were read using 96-well microplate ELISA reader (Dynatech MR 7000).
2. Estimation of plasma expression levels of microRNAs 146 and 155 using the qRT-PCR as described previously [15] according to the manufacturer’s instructions. The total plasma content of RNA was extracted using the miRNeasy Mini Kit (QIAGEN, Germany). Then, the relative quantity of both microRNAs was determined using the 2-step Real-time PCR by Maxima SYBR Green (QuantiTect SYBR Green PCR Kit: QIAGEN, Catalog no. 218,073, Str. 1–40,724) and the cDNA was synthesized using miScript II RT Kit (QIAGEN, Germany). The starting total RNA amount was mixed with buffers for reverse transcription reactions for quantization of miRNAs, and the recommended RNA input was incubated for 60 min at 37^o^C, and for 5 min at 95^o^C to inactivate the miScript RT. The mixture was then placed on ice and later diluted by 40 µl RNase-free water to the 10 µl reverse transcription reaction and mixed gently then briefly centrifuged and continued with real-time PCR using QuantiTect SYBR Green PCR Kit3 according to manufacturer’s instructions (SYBR). The PCR reaction mix was prepared in a total volume of 25 µl/tube (12.5 µl of 2x QuantiTect SYBR Green PCR Master Mix, 2.5 µl of 10x miScript Primer Assay, 2.5 ml of 10x miScript Universal Primer, Template cDNA up to 250 ng and RNase-free water). The real-time cycler was programmed using ABI 7900HT Fast Real-Time PCR System, (Applied Biosystem, Singapore). The amplification level was programmed with a denaturation step at 95 °C for 30 s, followed by 40 cycles at 95 °C for 10 s and 60 °C for 30 s, and the process is repeated for 40 cycles. The expression levels of microRNAs in each sample were determined after correction with the GADPH expression level. Controls were chosen as the reference samples, and fold changes in the levels of microRNAs were determined by the 2-^∆∆^CT (cycle threshold) method and expressed as fold change (FC) using Biosystems, USA) [16].

### The sequences of the used primers for the detection of the expression levels of the studied microRNA

**Table:** 

Items	Sequences
MiR-146a-F	5′-CAGCTGCATTGGATTTACCA-3′
MiR-146a-R	5′-GCCTGAGACTCTGCCTTCTG-3′
MiR-155-F	GGGGGTTAATGCTAATTGTGAT
MiR-155-R	AGTGCGTGTCGTGG
GAPDH-F	CCACCCATGGCAAATTCCATGGCA
GADDH-R	TCTAGACGGCAGGTCAGGTCCAC

### Statistical analysis

Results were analyzed using a t-test for independent means, One-way ANOVA for analysis of variance between groups, and a Chi-square test (X^2^ test) using the SPSS program (IBM, Ver. 22, 2015; Armonk, USA). Correlation, Regression, and the Receiver operating characteristic (ROC) curve analyses were used to determine the best predictor for outcomes. The significance of the differences between the obtained results was determined at a cutoff point of the probability of 0.05.

### Study outcomes

1. The primary outcome was the discriminative ability of estimated levels of plasma expression of genes of microRNAs for women who had *toxoplasma* infection among *toxoplasma* seronegative high-risk pregnant women.
2. The secondary outcome was the determination of the best early predictor for pregnancy outcome among the studied laboratory parameters.

## Results

**Fig. 1:**
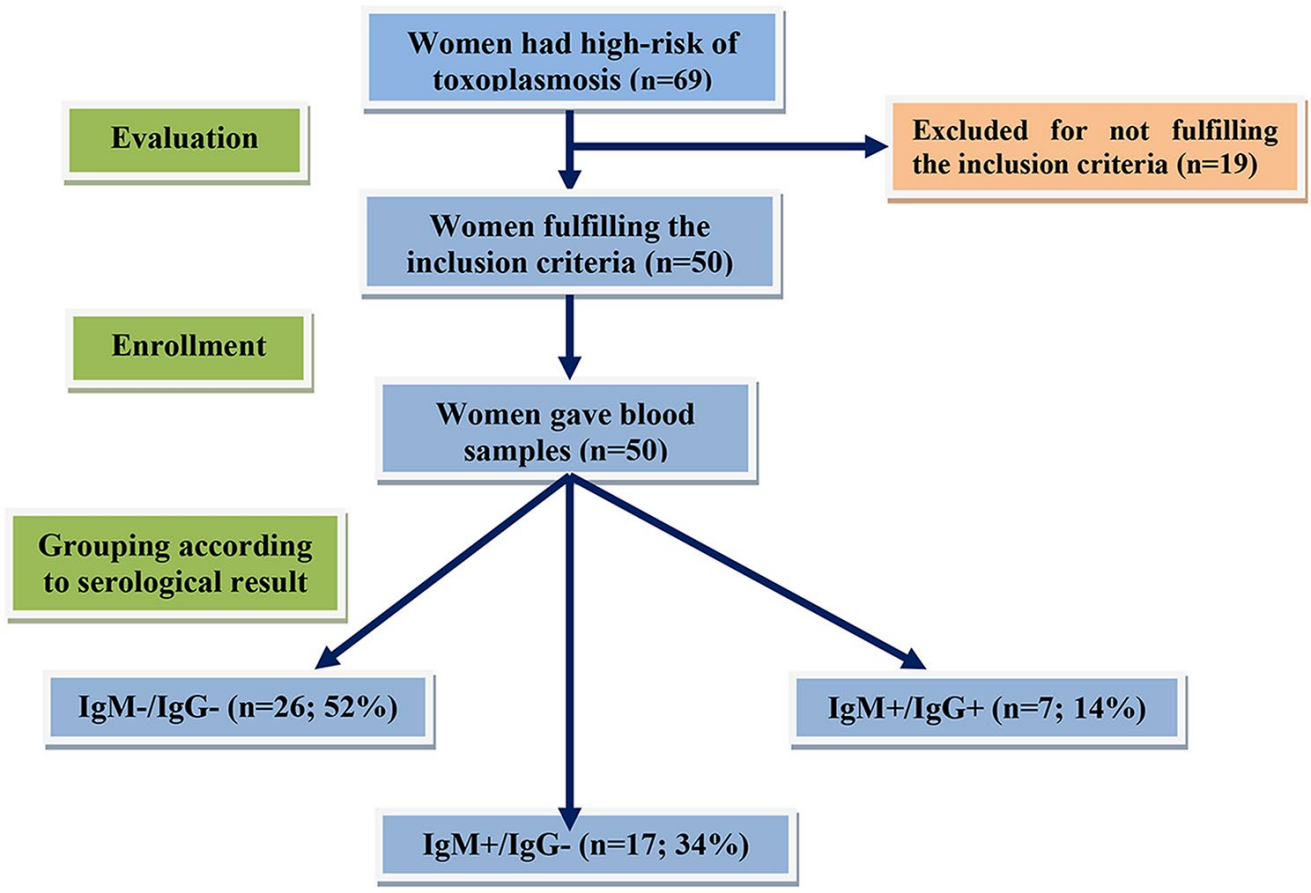
Study flow chart

The study included 50 newly pregnant women with a high risk of having toxoplasmosis after the exclusion of 19 pregnant women; 7 had a history of previous pregnancy loss due to *toxoplasma* infection, 4 women had manifest diabetes mellitus, 3 had essential hypertension, 3 women had uterine malformations and two patients had persistent urinary tract problems. ELISA evaluation of serum samples for *toxoplasma* antibody defined 26 women (52%) had negative toxoplasmosis IgM and IgG antibodies (A-group), serum samples of 17 women (34%) gave positive IgM antibody but were negative regarding toxoplasmosis IgG (B-group), while the samples of the remaining 7 women (14%) gave positive results concerning both IgM and IgG *toxoplasma* antibodies (C-group) (Fig. 1). Enrolment data of studied patients showed non-significant differences between groups (Table 1).

**Table 1:** Patients’ enrolment data

Data Group		A (*n* = 26)	B (*n* = 17)	C (*n* = 7)	*P*-value
Number (%)		26 (52%)	17 (34%)	7 (14%)	
Age (years)		28.6 ± 4.7	30.3 ± 5.4	29.7 ± 5.6	0.192
Body mass index (kg/m^2^)		30.6 ± 2.6	30.4 ± 3.2	31 ± 3.4	0.863
Gravidity	Primigravida	8 (30.8%)	5 (29.4%)	2 (28.6%)	0.979
	Multigravida	18 (69.2%)	12 (70.6%)	5 (71.4%)	
Area of residence	Urban	12 (46.2%)	7 (41.2%)	1 (14.3%)	0.206
	Rural	14 (53.8%)	10 (58.8%)	6 (85.7%)	
Employment	Housewives	7 (26.9%)	5 (29.4%)	3 (42.9%)	0.775
	Farmers	8 (30.8%)	9 (52.9%)	2 (28.6%)	
	Workers	5 (19.2%)	2 (11.8%)	1 (14.3%)	
	Officers	6 (23.1%)	1 (5.9%)	1 (14.3%)	
Presence of pets at home	Yes	5(19.2%)	4 (23.5%)	3 (42.9%)	0.176
	No	21 (80.8%)	13 (76.5%)	4 (57.1%)	
Relation to large animals	Yes	11 (42.3%)	8 (47.1%)	5 (71.4%)	0.382
	No	15 (57.7%)	9 (52.9%)	2 (28.6%)	
Eating raw food	Meat	2 (7.7%)	3 (17.6%)	1 (14.3%)	0.494
	Fish	4 (15.4%)	2 (11.8%)	0	0.814
	Fruits	12 (46.2%)	11 (64.7%)	3 (42.9%)	0.469
	Vegetables	19 (73.1%)	12 (70.6%)	4 (57.1%)	0.989
Drinking water	Tap	21 (80.8%)	12 (70.6%)	4 (57.1%)	0.073
	Wells	5 (19.2%)	5 (29.4%)	3 (42.9%)	
Hands’ cleaning habit before meals	Always	4 (15.4%)	3 (17.7%)	2 (28.5%)	0.204
	Frequent	10 (38.5%)	4 (23.5%)	1 (143%)	
	Infrequent	9 (34.6%)	6 (35.3%)	1 (14.3%)	
	No	3 (11.5%)	4 (23.5%)	3 (42.9%)	

Eighteen women (36%) completed their pregnancy course uneventfully without the development of any adverse outcomes, while 32 women (64%) had adverse pregnancy outcomes with weak significant (*P* = 0.049) difference in the frequency of complicated pregnancy according to seropositivity of the enrolled women. Differentially, the frequency of adverse pregnancy outcomes showed insignificant difference between the affected patients, irrespective of seropositivity. However, spontaneous abortion and preterm labor were the only pregnancy adverse outcomes affecting women of Group-C (IgM+/IgG+), while women of Group-A (IgM-/IgG-) had higher incidence of complications that mainly develop with continued pregnancy as gestational diabetes, gestational hypertension and premature rupture of the membrane, while the frequency of these complications for women of Group-B was low and absent for women of Group-C who had higher frequency of early pregnancy loss (Table 2).

**Table 2:** Patients’ distribution according to pregnancy outcomes

Pregnancy outcomes	Total (*n* = 50)	Groups			*P*-value
		A (*n* = 26)	B (*n* = 17)	C (*n* = 7)	
Uncomplicated	18 (36%)	10 (38.5%)	7 (41.2%)	1 (14.3%)	0.049
Complicated	32 (64%)	16 (61.5%)	10 (58.8%)	6 (85.7%)	
Spontaneous abortion/Early pregnancy loss	15 (30%)	7 (26.9%)	4 (23.5%)	4 (57.1%)	0.564
Preterm labor	7 (14%)	2 (7.7%)	3 (17.6%)	2 (28.6%)	0.491
Premature rupture of membrane	4 (8%)	3 (11.5%)	1 (5.9%)	0	0.801
Gestational diabetes mellitus	4 (8%)	3 (11.5%)	1 (5.9%)	0	0.180
Gestational hypertension	2 (4%)	1 (3.8%)	1 (5.9%)	0	0.619

Estimated plasma expression levels of Mir-146a and 155 were significantly higher in total patients and patients categorized according to their seropositivity in comparison to levels estimated in control women. Differentially, the plasma expression levels of both MicroRNAs were significantly higher in patients of the C-group in comparison to patients of other groups with non-significant differences between levels estimated in samples of patients of other groups (Table 3).

**Table 3:** Plasma expression levels of MiR-146a and MiR-155 estimated in the studied patients

Variate Groups		Control (*n* = 50)	Total patients (*n* = 50)	A (*n* = 26)	B (*n* = 17)	C (*n* = 7)
MiR-146a	Expression level	0.58 ± 0.22	1.08 ± 0.32	1 ± 0.29	1.09 ± 0.25	1.38 ± 0.44
	P vs. control group		< 0.001	< 0.001	< 0.001	< 0.001
	P vs. IgM^−^/IgG^−^ group				0.309	0.0096
	P vs. IgM^+^/IgG^−^ group					0.029
MiR-155	Expression level	0.97 ± 0.24	2.35 ± 1.06	2.36 ± 0.9	1.91 ± 0.95	3.36 ± 1.26
	P vs. control		< 0.001	< 0.001	< 0.001	< 0.001
	P vs. IgM^−^/IgG^−^ group				0.129	0.025
	P vs. IgM^+^/IgG^−^ group					0.005

**Fig. 2:**
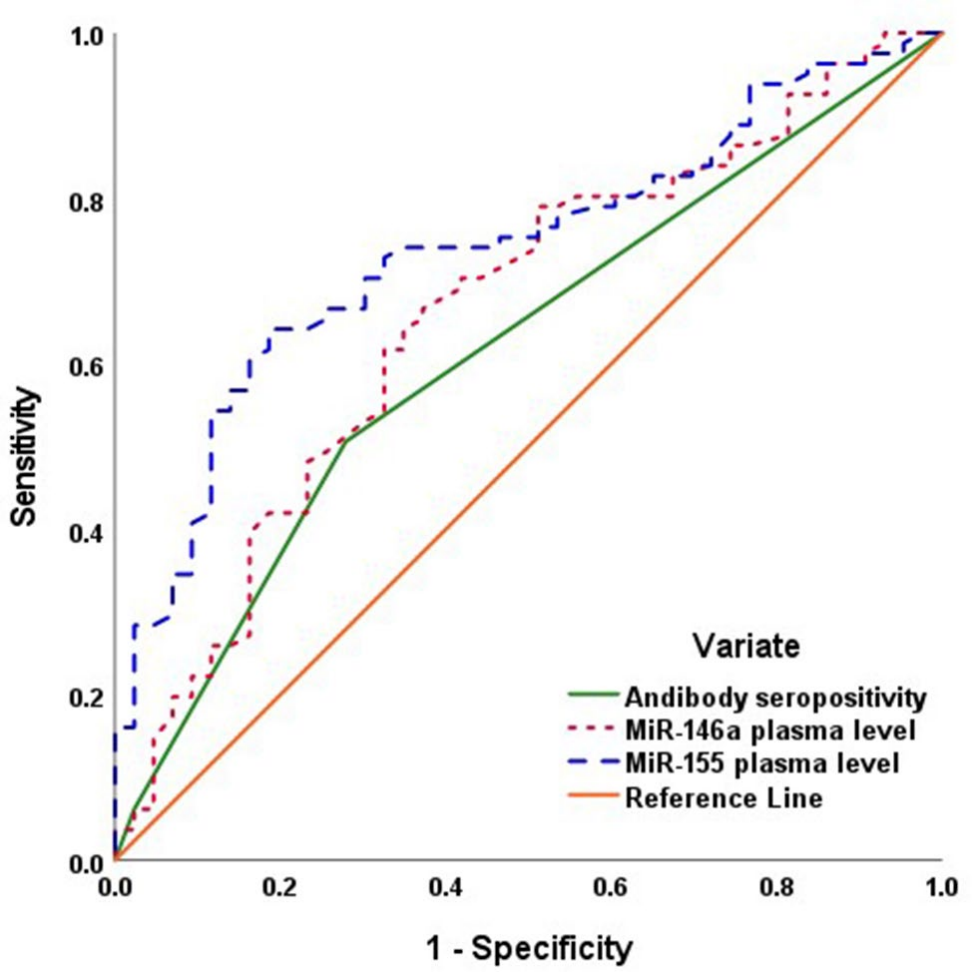
ROC curve analysis for laboratory findings as predictors for pregnancy outcomes

**Fig. 3:**
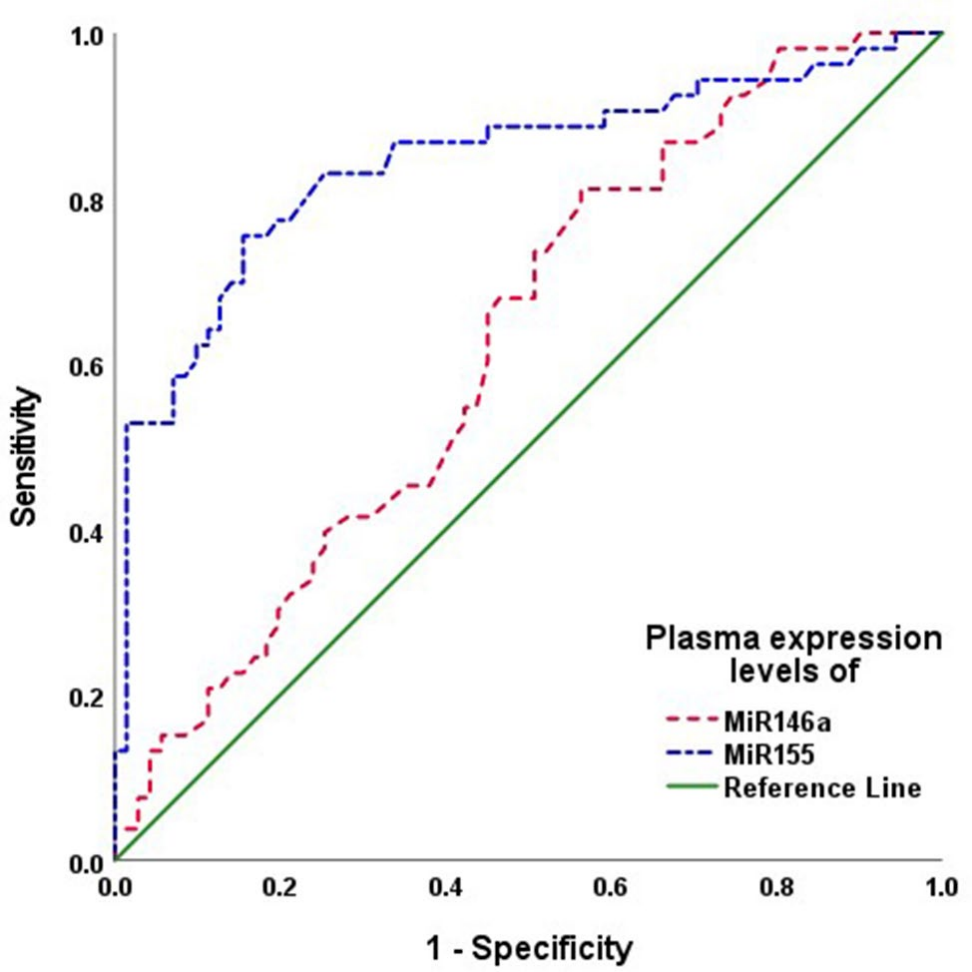
ROC curve analysis for plasma expression levels of microRNAs as predictor for Toxoplasmosis in seronegative patients

The incidence of adverse pregnancy outcomes showed positive significant correlations with the seropositivity for *toxoplasma* antibodies and high expression levels of MiR-146a and MiR-155. The ROC curve analysis and Regression analysis assured these correlations and defined high plasma level of MiR-155 as the highly significant predictor for oncoming pregnancy complications (Fig. 2). As predictors for the presence of toxoplasmosis despite the negative serology, both microRNAs could identify these patients as shown by ROC curve but the difference in AUC was significant (*P* < 0.001) in favor of MiR-155 (Table 4; Fig. 3).

**Table 4:** Statistical analyses of the results of the studied laboratory parameters

Variate	Parameters Statistical analyses		Antibody seropositivity	Plasma level of MiR-146a	Plasma level of MiR-155
Adverse pregnancy outcome	Pearson’s correlation analysis	r	0.215	0.260	0.382
		P	0.017	0.003	< 0.001
Prediction of oncoming adverse pregnancy outcome	ROC curve analysis	AUC	0.616	0.662	0.739
		Std. Error	0.052	0.051	0.045
		P	0.033	0.003	< 0.001
		95% CI	0.514–0.718	0.561–0.763	0.651–0.827
	Regression analysis	β	0.061	0.147	0.382
		P	0.584	0.099	< 0.001
Prediction of toxoplasmosis among seronegative women	ROC curve analysis	AUC	-	0.626	0.843
		Std. Error		0.050	0.038
		P		0.017	< 0.001
		95% CI		0.528–0.723	0.769–0.918
	Paired-sample AUC difference	AUC-difference		0.218	
		Std. Error		0.269	
		P		< 0.001	
		95% CI		0.106–0.329	

**Fig. 4:**
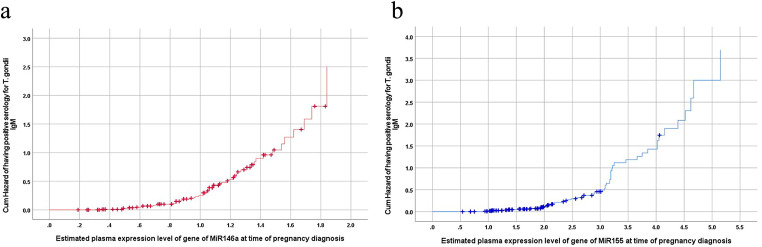
(**a**) Kaplan-Meier analysis for plasma level of MiR-146a gene as predictor for *T. gondii* infection in seronegative women. (**b**) Kaplan-Meier analysis for plasma level of MiR-155 gene as predictor for *T. gondii* infection in seronegative women

**Fig. 5:**
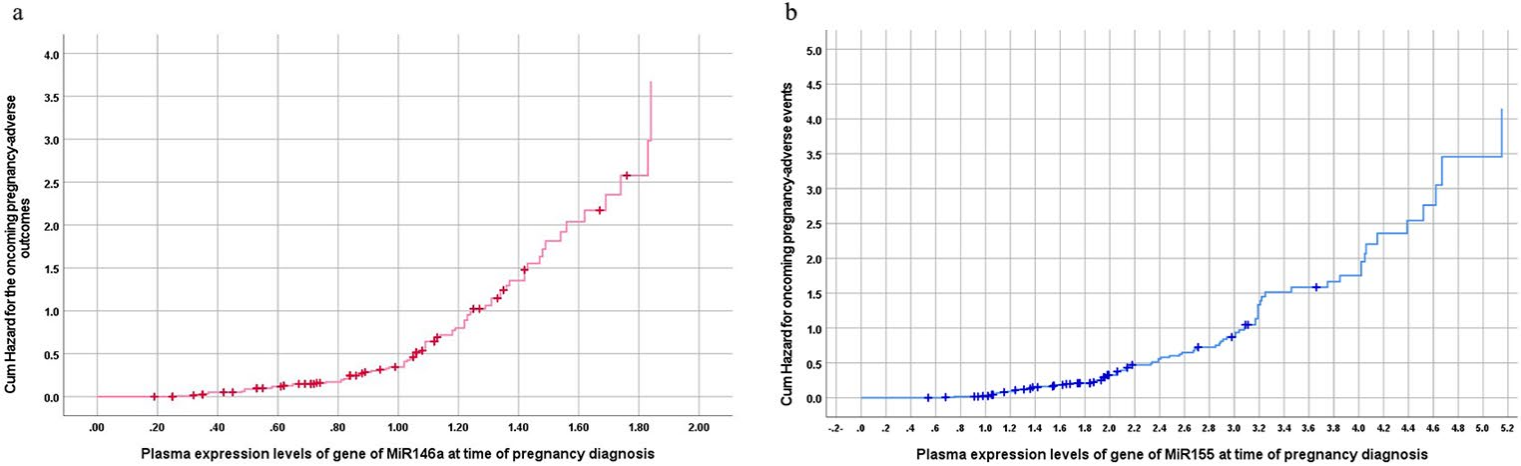
(**a**) Kaplan-Meier analysis for plasma level of MiR-146a gene as predictors for the cumulative risk of the oncoming adverse pregnancy outcomes. (**b**) Kaplan-Meier analysis for plasma level of MiR-155 gene as predictors for the cumulative risk of the oncoming adverse pregnancy outcomes

Further, Kaplan-Meier regression analysis defined the cumulative risk of having toxoplasmosis despite negative serology as 25% at plasma levels of MiR-146a of 1 and at the level of 1.2 the cumulative risk was doubled to 50% (Fig. 4a). For plasma levels of MiR-155, the cumulative risk was 25% and 50% at expression levels of 2.5 and 3, respectively (Fig. 4b). According to Kaplan-Meier regression analysis, the cumulative risk of oncoming pregnancy adverse outcomes was 50% at a cutoff point of expression of genes of MiR-146a and MiR-155 of 1.05 and 2.2, respectively and was doubled at the plasma expression level of 1.22 for MiR-146a and 3.1 for MiR-155 (Fig. 5a&b).

## Discussion

Among the studied women susceptible to *toxoplasma* infection, the rate of seropositivity was 48%, while the reported incidence of complicated pregnancy was 64% with a frequency of 1.64 complications/patient, irrespective of the serological diagnosis. These findings coincided with Kazemi et al. [17] who detected an abortion rate of 32% among IgG seropositive women and concluded that *toxoplasma* infection could be considered the most significant cause of abortion. Also, Çakırca et al. [18] reported a prevalence and acute infection rates of toxoplasmosis of 46.2% and 4% among pregnant women with an incidence of the mother-to-child transmission rate of 5%. Further, Hassan et al. [19] detected IgG seropositivity to *T. gondii* in 45.2% of their series of pregnant women and found the rate of seropositive women changed in ascending direction with pregnancy progress from 44.2 to 67.7% in the 1st and 3rd trimesters, respectively.

The reported frequency of pregnancy complications was higher among women who had seronegativity for both IgM and/or IgG, thus the reliance on the detection of Ig for *toxoplasma* could not predict the outcome of pregnancy. In support of this assumption, the correlation between seropositivity and adverse pregnancy outcome was weakly significant and ROC curve analysis defined seropositivity as a weak significant predictor, while regression analysis excluded serology as a predictor for pregnancy outcomes. These findings point to the need for supplemental parameters to *toxoplasma* serology to assure the negativity, in line with these findings, Mihu et al. [20] detected a high prevalence of seropositivity for *T. gondii* among females of reproductive age, but documented that detection of *T. gondii* IgA antibodies may improve the rate for the detection of recently acquired toxoplasmosis. Moreover, Hassan et al. [19] concluded that the results of routine serological screening for *T. gondii* must be integrated with antenatal services to define the potential *toxoplasma* infection.

The results of this work showed high diagnostic performance of estimated plasma expression levels of MiR-146a and 155 genes than serology for prediction of adverse pregnancy outcomes in women susceptible to toxoplasmosis with significant AUC (*P* = 0.003 & <0.001, respectively), but Regression analysis defined high plasma levels of MiR-155 as the significant predictor, while excluded MiR-146a. Further, the ROC curve showed significant AUC for MiR-146a and 155 (*P* = 0.017 & <0.001) as markers for discriminating women had toxoplasmosis despite the negative serology and Paired-sample AUC difference defined as significant (*P* < 0.001) difference in favor of MiR-155 as a discriminating parameter.

These data spotlight the role of microRNAs in pathogenesis, progression or control of toxoplasmosis disease and the possibility of using these microRNAs as biomarkers for diagnosis in uncomplicated and complicated cases. In support of these assumptions, experimentally, Antil et al. [21] identified novel autocrine/paracrine signaling factors that could be associated with host response modulated by *T. gondii* through 74 cores differentially regulated miRNAs and their 319 high-confidence mRNA targets. Also, Xie et al. [7] detected dysregulated non-coding RNAs, 88 common differentially expressed miRNAs and 120 novel differentially expressed PIWI-interacting RNAs in both serum and urine samples of rabbits infected by *T. gondii* oocysts. Thereafter, Zheng et al. [22] using a model of dogs infected by *T canis* detected the expression of 3 and 25 miRNAs in serum at 24-h and 10 days post-infection and concluded that miRNAs have a regulatory role in definitive hosts after *T. canis*.

Regarding the studied microRNAs; Cannella et al. [23] detected expression of MiR-146a and 155 in the brains of mice challenged with *toxoplasma* and found miR-146a has modulatory action on the infection through the rhoptry kinase; additionally, miR-146a deficiency induced better control of parasite burden in the gut and its early brain dissemination and concluded that MiR-146a and 155 are immunomodulatory for *toxoplasma* infected cells. Thereafter, da Cruz et al. [24] detected overexpression of miR-146a, 155, 21, 29c and 125b in serum-derived extravascular vesicles (EVs) from patients who had cerebral and gestational toxoplasmosis. Also, Meira-Strejevitch et al. [25] detected significantly higher expression levels of miR-155 and an insignificantly higher level of miR-146a in patients of ocular than in patients of asymptomatic toxoplasmosis. Recently, Zou et al. [26] detected 177 and 77 differentially expressed miRNAs in livers at the acute and chronic *T. gondii* infection stages, respectively and miR-146a and − 150 were associated with liver immunity and pathogenesis of toxoplasmosis and Wang et al. [27] using an animal model of mice *T. gondii* infection, detected upregulation of MiR-146a.

Using cell culture, Yi-Hong et al. [28], detected upregulation of miR-155 by > 4-fold in *T. gondii* infected macrophages with enhanced production of mRNA of inducible nitric oxide (NO) synthase and interleukin-12 (IL-12) by PCR and increased levels of NO and IL12 by ELISA. In an animal model of mice infected by *T. gondii*, Xu et al. [29] found lack of miR-155 led to increased parasite burden, decreased animal survival, reduced innate and adaptive immune responses with decreased pro-inflammatory mediators, and worsened CD8 + T cell exhaustion and concluded that miR-155 is a critical immune regulator for the control of *T. gondii* infection and could be used as molecular target for boosting immunity against *T. gondii*. In another study, using mice liver tissue infected by virulent and avirulent strains of *T. gondii*, El-Sayad et al. [30] detected significant overexpression of miRNA-155 with a significant reduction of butyrylcholinesterase in all infected cells, but were maximal in cells infected by virulent strains especially at 7-d after infection and concluded that miRNA-155 and butyrylcholinesterase play a role in regulating host-parasite interaction in toxoplasmosis. Further, Quiarim et al. [31] found *T. gondii*-derived EVs can promote host-parasite interactions, modulate host immune responses, carry virulent factors and cause an imbalance in cellular immune response mostly through overexpression of miR-155, 125b and 423.

Recently, Jiang et al. [32] detected upregulation of miR-155-5p expression in exosomes secreted by immortalized murine dendritic cells created by transducing bone marrow isolate that are infected by *T. gondii*, these exosomes and miR-155 stimulate host pro-inflammatory immune responses, activation of nuclear factor (NF)-κB the pathway leading to inhibition of *T. gondii* tachyzoite proliferation in infected cells. Also, Maia et al. [33] reported that miRNAs participate in the modulation of the cellular immune response against *T. gondii* especially miR-155, 29c and 125b, which were overexpressed in mice immunized with *T. gondii*-released EV, acutely and chronically infected mice than normal mice.

## Conclusion

Toxoplasmosis is associated with a high incidence of pregnancy complications especially abortion and early pregnancy loss. The incidence of pregnancy complications is high for seropositive or seronegative women at high risk of toxoplasmosis. Estimated plasma levels of gene expression of MiR-146a and 155 might identify women liable to develop complications and differentiate seronegative women vulnerable to having *T. gondii* infection. The diagnostic performance of estimated levels of MiR-155 was higher than MiR-146a and might be the best predictor for pregnancy outcomes of women at high risk of toxoplasmosis.

### Limitations

Ruling out the presence of co-infection is limitation of the current study.

## Data Availability

The datasets used and/or analysed during the current study available from the corresponding author on reasonable request.

## Abbreviations

MiR-: microRNA
*T. gondii*: *Toxoplasma gondii*
IgG: Immunoglobulin G
IgM: Immunoglobulin M
ANC: Antenatal Care Unit
BMI: Body mass index
SBP: Systolic blood pressures
DBP: Diastolic blood pressures
FBG: Fasting blood glucose
PPBG: Postprandial blood glucose
IR: Insulin resistance
qRT-PCR: Quantitative reverse transcriptase polymerase chain reaction
CT: Cycle threshold
FC: Fold change
ROC: Receiver operating characteristic
